# Evidence for a chromosome origin unwinding system broadly conserved in bacteria

**DOI:** 10.1093/nar/gkab560

**Published:** 2021-07-01

**Authors:** Simone Pelliciari, Mei-Jing Dong, Feng Gao, Heath Murray

**Affiliations:** Centre for Bacterial Cell Biology, Biosciences Institute, Newcastle University, Newcastle Upon Tyne NE2 4AX, UK; Department of Physics, School of Science, Tianjin University, Tianjin, China; Frontiers Science Center for Synthetic Biology and Key Laboratory of Systems Bioengineering (Ministry of Education), Tianjin University, Tianjin, China; Department of Physics, School of Science, Tianjin University, Tianjin, China; Frontiers Science Center for Synthetic Biology and Key Laboratory of Systems Bioengineering (Ministry of Education), Tianjin University, Tianjin, China; Centre for Bacterial Cell Biology, Biosciences Institute, Newcastle University, Newcastle Upon Tyne NE2 4AX, UK

## Abstract

Genome replication is a fundamental requirement for the proliferation of all cells. Throughout the domains of life, conserved DNA replication initiation proteins assemble at specific chromosomal loci termed replication origins and direct loading of replicative helicases (1). Despite decades of study on bacterial replication, the diversity of bacterial chromosome origin architecture has confounded the search for molecular mechanisms directing the initiation process. Recently a basal system for opening a bacterial chromosome origin (*oriC*) was proposed (2). In the model organism *Bacillus subtilis*, a pair of double-stranded DNA (dsDNA) binding sites (DnaA‐boxes) guide the replication initiator DnaA onto adjacent single-stranded DNA (ssDNA) binding motifs (DnaA‐trios) where the protein assembles into an oligomer that stretches DNA to promote origin unwinding. We report here that these core elements are predicted to be present in the majority of bacterial chromosome origins. Moreover, we find that the principle activities of the origin unwinding system are conserved *in vitro* and *in vivo*. The results suggest that this basal mechanism for *oriC* unwinding is broadly functionally conserved and therefore may represent an ancestral system to open bacterial chromosome origins.

## INTRODUCTION

**Figure 1.**
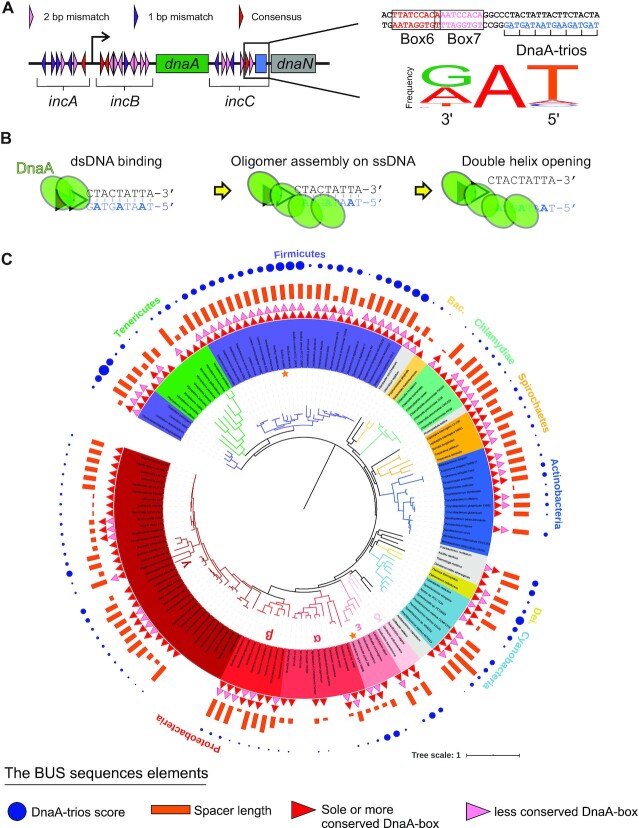
Bioinformatic analysis identifies putative BUS sequence elements within *oriC* throughout the bacterial domain. (**A**) Schematic representation of *B. subtilis oriC* highlighting the sequence elements of the BUS (DnaA-boxes #6/#7 and DnaA-trios). The Weblogo shows that the DnaA-trio motif is degenerate(22). (**B**) Simplified illustration of BUS activity. DnaA binds the two DnaA-boxes, from which it assembles into a oligomer specifically on the DnaA-trios sequence to stretch and separate the two strands. (**C**) Phylogenetic analysis displaying the BUS sequence elements. Note that this phylogenetic tree is modified based on the tree of life (iTOL), and we retain the same bacteria in the tree and DoriC 6.5. The tracks from outside to inside correspond to DnaA-trio (blue dot, size reflects score), the spacer (orange bar, reflecting the length), and the DnaA-boxes (solid triangles, red indicating the highest sequence conservation). Each phylum is marked outside of the tree. The five classes in Proteobacteria are marked inside of the tree by Greek letters. *B. subtilis* and *H. pylori* are marked by stars.

In bacteria, the toroid hexameric helicases that drive bidirectional DNA replication from the chromosome origin are loaded around ssDNA (3). To permit helicase ssDNA access, bacterial chromosome origins are opened by the ubiquitous initiation protein, DnaA (4). DnaA is a multifunctional enzyme composed of four distinct domains that act in concert during DNA replication initiation (5). Domain IV contains a helix-turn-helix dsDNA binding motif that specifically recognizes 9 bp asymmetric sequences called ‘DnaA-boxes’ (highest affinity 5′-TTATCCACA-3′) (Figure 1A) (6–9). Domain III is composed of the AAA+ motif that can assemble into an ATP-dependent right-handed helical oligomer (Supplementary Figure S1) (10–12). A critical residue of this domain involved in oligomer formation is the invariant ‘arginine finger’ that makes contact with the γ-phosphate of ATP bound by the adjacent DnaA protein (11). Domain III also contains the residues required for a DnaA oligomer to interact with ssDNA, particularly a conserved hydrophobic amino acid within the ‘Initiator Specific Motif’ (ISM) (Supplementary Figure S1) (13,14). Domain II tethers domains III-IV to domain I, which acts as an interaction hub that facilitates DnaA oligomerization (15) and loading of the replicative helicase (16).

Bacterial replication origins encode information that promotes specific opening of the DNA duplex by DnaA (17,18). Typically, *oriC*s contain multiple DnaA-boxes flanked by the site of DNA unwinding (7,19). However, bacterial chromosome origin architecture is diverse, which has precluded the recognition of core sequence features (20,21). Recent work identified a DnaA-dependent system that promotes *oriC* opening in the model organism *Bacillus subtilis*, which is composed of two DnaA-boxes (#6 and #7) adjacent to a repeating trinucleotide motif termed ‘DnaA-trios’ (consensus 3′-GAT-5′) (Figure 1A) (2,22). Here DnaA binding to DnaA-box#6/7 (dsDNA via domain IV) acts as a platform directing the assembly of additional DnaA^ATP^ monomers into an oligomer that engages the DnaA-trios (ssDNA via domain III) (Figure 1B) (2,22). The interaction between a DnaA^ATP^ oligomer and DnaA-trios is thought to promote unwinding of the DNA duplex through a ssDNA stretching mechanism (2,13). For simplicity here, we will refer to this as a ‘basal unwinding system’ (BUS). In this study we investigated whether the *oriC* unwinding system identified in *B. subtilis* is conserved in other bacterial species.

## MATERIALS AND METHODS

### Media and chemicals

Nutrient agar (NA, Oxoid) was used for routine selection and maintenance of *Escherichia coli* strains. For experiments, *E. coli* cells were grown using Luria–Bertani (LB) medium. Supplements were added as required: 100 μg/ml ampicillin. *H. pylori* 26695 strain was purchased from National Collection of Type Cultures of Public Health England and maintained on Columbia Blood Agar (CBA) plates containing 5% defibrinated Sheep Blood (Thermo Fisher Scientific) in microaerofilic chambers (Oxoid) in which the atmosphere was maintained with CampyGen 2.5L Sachet (Oxoid). Supplements were added as required: 5 μg/ml chloramphenicol, 10 μg/ml kanamycin. Unless otherwise stated, all chemicals and reagents were obtained from Sigma-Aldrich.

### *Helicobacter pylori* transformation

To generate *H. pylori* mutants, the recipient strains were revitalized from glycerol stocks onto fresh CBA plates at 37°C. Colonies of each strain were serially expanded on CBA plates until a confluent layer was achieved. These cells were compacted into a sphere and left for 5 h at 37°C to promote natural development of genetic competency. The cells mass was then transferred onto a pre-warmed CBA plate and left for 1h at 37°C. Five μg of DNA in a volume of <10 μl was gently added atop the sphere and mixed using an inoculating loop. The cells were then compacted and incubated overnight at 37°C. Finally the cells were then spread onto a prewarmed CBA plate containing antibiotic and incubated at 37°C. Transformants generally appeared after 4–5 days.

### Protein expression and purification

The *dnaA* gene from each species was amplified by PCR using either genomic DNA or a synthetic gene (GenScript) and cloned into pSP015 containing a His_14_-SUMO-FLAG_3_ tag. Plasmids were amplified in DH5α and then transformed into BL21(DE3)-pLysS. Strains were grown in LB medium at 37°C. At *A*_600_ 0.6, 1 mM IPTG was added and cultures were incubated for 4h at 30°C. For *B. subtilis* DnaA, cells were harvested by centrifugation at 7000 g for 20 min, resuspendend in 40 ml of Ni^2+^ Binding Buffer (30 mM HEPES [pH 7.6], 250 mM potassium glutamate, 10 mM magnesium acetate, 20% sucrose, 30 mM imidazole) containing 1 EDTA-free protease inhibitor tablet (Roche #37378900) and flash frozen in liquid nitrogen. Cell pellet suspensions were thawed and incubated with 0.5 mg/ml lysozyme on ice for 1h. Cells were disrupted by sonication (5 cycles of 3 min at 30 W with 2 s pulses/rests interval). To remove cell debris the lysate was centrifuged at 24 000 g for 30 min at 4°C, then passed through a 0.2 μm filter for further clarification. All the further purification steps were performed at 4°C using a FPLC with a flow rate of 1 ml/min.

The clarified lysate was applied to a 1 ml HisTrap HP column (GE), washed with 10 ml Ni^2+^ High Salt Wash Buffer (30 mM HEPES [pH 7.6], 1 M potassium glutamate, 10 mM magnesium acetate, 20% sucrose, 30 mM imidazole) and 10 ml of 10% Ni^2+^ Elution Buffer (30 mM HEPES [pH 7.6], 250 mM potassium glutamate, 10 mM magnesium acetate, 20% sucrose, 1 M imidazole). Proteins were eluted with a 10 ml linear gradient (10–100%) of Ni^2+^ Elution Buffer. Fractions containing the protein were applied to a 1 ml HiTrap Heparin HP affinity column (GE) equilibrated in H Binding Buffer (30 mM HEPES [pH 7.6], 100 mM potassium glutamate, 10 mM magnesium acetate, 20% sucrose). Proteins were eluted with a 20 ml linear gradient (20–100%) of H Elution Buffer (30 mM HEPES [pH 7.6], 1 M potassium glutamate, 10 mM magnesium acetate, 20% sucrose). Fractions containing DnaA (usually 3 ml total) were pooled and digested overnight with 10 μl of 10 mg/ml His_14_-Tev-SUMO protease (23).

The digested reaction was applied to a 1 ml HisTrap HP column to capture non-cleaved monomers, His_14_-SUMO-tag and His-_14_-TEV-SUMO protease. Cleaved DnaA proteins were collected in the flow-through and their purity was confirmed using SDS-PAGE. Glycerol was added (20% final) and proteins aliquots were flash frozen in liquid nitrogen before being stored at -80°C.

The purification of DnaA homologs from *Listeria monocytogenes*, *Staphylococcus aureus* and *Enterococcus faecalis* was performed as described above with the exception that samples were diluted to a final potassium glutamate concentration of 100 mM before being applied to the heparin column.

The purification of DnaA homologs from *Synechococcus elongatus*, *Helicobacter pylori* and *Deinococcus radiodurans* was performed as described above with the following exceptions. First, the Heparin column was omitted. Second, before applying the digested proteins to a HisTrap HP column samples were diluted to a final imidazole concentration of 50 mM.

### Fluorescent ATP analog binding assay

Proteins (8 μM final) were incubated with 0.16 mM of (2′-(or-3′)-*O*-(trinitrophenyl) adenosine triphosphate (TNP-ATP) (Thermo Fisher Scientific), in 50 μl of Binding Buffer (10 mM HEPES–KOH [pH 8], 100 mM potassium glutamate, 5 mM magnesium acetate). A reaction containing 8 μM of lysozyme was used as a negative control. All reactions were performed for 10 min at 25°C in a black flat-bottom polystyrene 96-well plate (Costar #CLS3694). TNP-ATP was excited at 410 nm and fluorescence emission was detected between 500–650 nm using a plate reader (CLARIOstar Plus, BMG Labtech).

### DNA scaffolds

DNA scaffolds were prepared in a 20 μl reaction with each oligonucleotide (10 μM) mixed in Oligo Annealing Buffer (30 mM HEPES–KOH [pH 8], 100 mM potassium acetate and 5 mM magnesium acetate). The samples were heated in a PCR machine to 95°C for 5 min and then cooled 1°C/min to 20°C before being held at 4°C prior to dilution to 1 μM in Oligo Annealing Buffer and storage at –20°C. Scaffolds separated by polyacrylamide gel electrophoresis (containing Cy3 and Cy5 fluorophore) were imaged with a Typhoon FLA 9500 laser scanner (GE Healthcare) at 400 V with excitation at 532 or 635 nm and emission filter LPR (580BP or 665LP respectively). Images were processed using Fiji (https://doi.org/10.1038/nmeth.2019). DNA probes that contain both Cy5 and Black Hole Quencher-2 (BHQ-2) were analysed using a plate reader (CLARIOstar Plus, BMG Labtech) with pre-set filters for Cy5 fluorophore (Excitation 610-30, Dicroic 637.5, Emission 675-50) setting the gain at 2800.

In an attempt to maintain the *T*_m_ of oligonucleotides, DnaA-trios were mutagenized while maintaining the base content (see Supplementary Table S3). To measure the melting temperature of WT and DnaA-trios^MUT^ substrates related to each organism, we prepared a 20 μl reaction containing 0.4 μM of probe in different reaction buffer (see below the reaction buffer associated with each probe). A melting curve from 20 to 95°C was then performed on the reaction using a Rotor-GenQ qPCR machine (QIAgen) with 563 excitation filter and 610 high-pass emission filter (gain:10). The curves obtained and the melting value of each peak are reported in Supplementary Table S3.

### Black hole quencher DNA strand separation assay (BHQ-SSA)

Each DNA scaffold containing one oligonucleotide with BHQ2, one with Cy5 and one unlabelled (12.5 nM final concentration) was diluted in an appropriate buffers (see below). Reactions were performed using a flat-bottom black polystyrene 96-well plate (Costar #CLS3694). All reactions were made in triplicate and fluorescence was detected every minute for a total of 90 min. For all reactions a negative control without protein was performed (background). At each timepoint the average background value is subtracted from the experimental value, thus reporting the specific DnaA activity on a single substrate.

**Figure 2.**
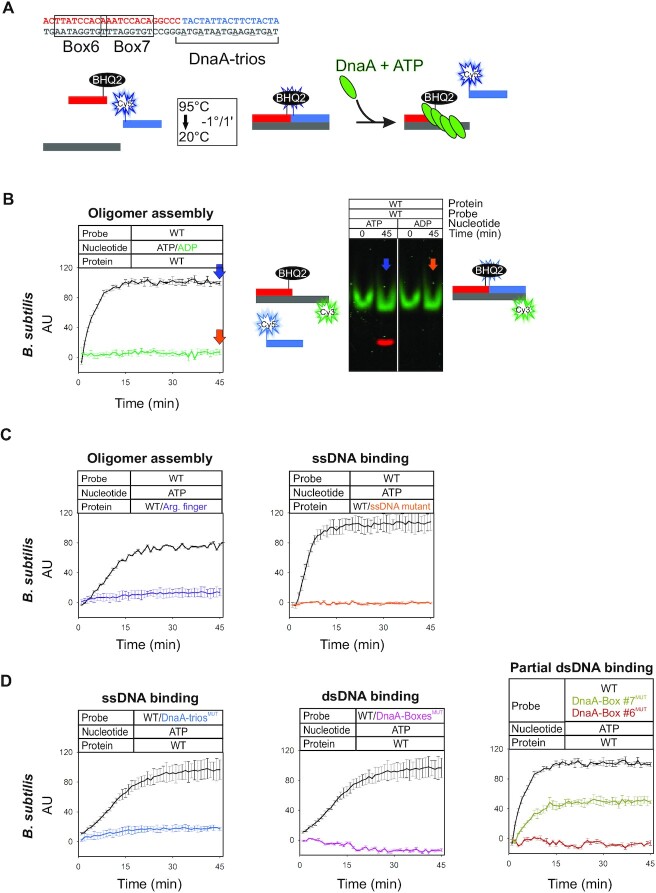
Specific BUS unwinding assay using *B. subtilis* DnaA. (**A**) DNA scaffold sequences for *B. subtilis* and schematic representation of the Black Hole Quencher strand separation assay (BHQ-SSA). (**B**) BHQ-SSA using *B. subtilis* DnaA indicates that ATP is required for scaffold unwinding. A polyacrylamide gel showing the specific separation of the Cy5-labelled oligonucleotide from the scaffold. (**C**) BHQ-SSA using *B. subtilis* DnaA proteins indicates that the arginine-finger (DnaA^R264A^, in violet) and the ssDNA binding residue (DnaA^I190A^, in orange) are required for scaffold unwinding. (**D**) BHQ-SSA using *B. subtilis* DnaA indicates that both DnaA-boxes and DnaA-trios are required for scaffold unwinding. Data represent the mean and standard deviation from three independent experiments. Y-axis shows fluorescence arbitrary unit (AU).

To directly visualize the reaction products (Figure 2B), 20 μl samples were removed and added to 3.7 μl of stop solution containing 250 nM unlabelled competitor (same sequence as the oligonucleotide containing Cy5), 0.4% SDS and 1 mg/ml proteinase K. The reactions were stored on ice and then loaded onto a 4% polyacrylamide (19:1) gel and run at 100 V for 3 h in buffer containing 45 mM Tris, 45 mM boric acid and 5 mM magnesium chloride.

For *B. subtilis*, *S. aureus* and *E. faecalis* the reaction buffer was 10 mM HEPES-KOH (pH 8), 100 mM potassium glutamate, 2 mM magnesium acetate, 30% glycerol, 10% DMSO and 1 mM nucleotide (ADP or ATP).

For *L. monocytogenes, S. elongatus*, *H. pylori* and *D. radiodurans* the buffer used was 10 mM HEPES-KOH (pH 8), 100 mM potassium glutamate, 2 mM magnesium acetate, 30% glycerol and 1 mM nucleotide (ADP or ATP).

All the reactions were prepared on ice to ensure the stability of the DNA probe, then allowed to equilibrate to the reaction temperature (20°C for *L. monocytogenes*, *E. faecalis* and *S. aureus*; 25°C for *B. subtilis*, *S. elongatus* and *H. pylori*; 32°C for *D .radiodurans*). DnaA protein was added to the final concentration of 130 nM for *B. subtilis* and *D. radiodurans* and 650 nM for *L. monocytogenes*, *E. faecalis*, *S. aureus*, *S. elongatus* and *H. pylori*. For *B. subitlis* on DnaA-Box #7^MUT^ and DnaA-Box #6^MUT^ protein was added at the final concentration of 650 nM to allow displacement of all the probes.

### Immunoblotting

Purified protein eluted from 1 ml HiTrap Heparin HP affinity column (GE) was fixed in NuPAGE LDS sample buffer (Thermo Fisher Scientific) at 98°C for 5 min and complexes were resolved by running 8 μl from each sample on a NuPAGE Novex 4–12% Tris-acetate gel (Thermo Fisher Scientific). Proteins were transferred to a PVDF membrane using Turbo-Blot transfer apparatus and Trans-Blot TurboTM Midi PVDF Transfer Packs (Bio-Rad). The membrane was blocked with PBS + 5% milk for 1 h at room temperature then incubated with a PBS + 1% milk solution containing a 1:2000 monoclonal anti-FLAG antibody (Sigma-Aldrich #F3165) for 1 h at room temperature. The membrane was washed three times with PBS + 0.05% Tween-20 and then incubated with PBS + 1% milk solution containing a 1:10 000 anti-mouse horseradish peroxidase conjugated secondary antibody (Sigma-Aldrich #A9044). The membrane was washed three times with PBS + 0.05% Tween-20 and then incubated for 5 min with Pierce ECL Plus substrate (Thermo Scientific). Chemiluminescence was detected using an ImageQuant LAS 4000 imager (GE Healtcare). Images were processed using Fiji (https://doi.org/10.1038/nmeth.2019).

### Protein crosslinking

DNA scaffolds were prepared by mixing each oligonucleotide (50 nM final concentrations) in 10 mM HEPES–KOH (pH 7.6), 100 mM NaCl and 1 mM EDTA, heating to 98°C for 5 min in a heat block and then slowly cooling to room temperature. DnaA^CC^ proteins (250 nM) were combined with a DNA scaffold (25 nM) in 10 mM HEPES–KOH (pH 8), 100 mM potassium glutamate, 100 mM sodium chloride, 10 mM magnesium acetate, 25% glycerol, 0.01% Tween-20 and 2 mM nucleotide (ADP or ATP). Reactions were incubated at 37°C for 6 min, then 2.5 μl of 20 mM bismaleimidoethane (BMOE) (4 mM final) (Thermo Fisher Scientific #803561) was added to crosslink DnaA^CC^ proteins. After 6 min the reaction was quenched by addition of 5 μl of 200 mM cysteine (60 mM final) for 10 min. Samples were fixed in NuPAGE LDS sample buffer (Thermo Fisher Scientific) at 98°C for 5 min and complexes were resolved and visualized by running 8 μl from each reaction following the method described above. The interpretation of the crosslinked species was based on their migration relative to molecular weight markers. All experiments were independently performed at least thrice, and representative data are shown.

### The program to search for the BUS sequence

A program was designed to identify the BUS sequences in bacterial replication origins based on the pattern of experimental and predicted unwinding regions (22). During the training process, the program was adjusted to output the same predicted sequences as those identified by previous analysis of twenty *oriC*s, in order to generate the parameters and rules required by the program (Supplementary Table S1 and Figure S2). In general, the BUS sequence consists of three parts: DnaA-box, spacer and repeating DnaA-trios motifs. The determined parameters of the three parts during the training process are presented as follows:

1. *Standard motif of the DnaA-box*. If the species has a divergent DnaA-box in DoriC (21,24), the standard motif of DnaA-box was the species-specific one, whereas in the other cases it was the *E.coli* highest affinity DnaA-box motif (5′-TTATCCACA-3′).
2. *Mismatches of the predicted DnaA-box*. If the length of the standard motif is 9 bp and there is only one DnaA-box present, then the maximum mismatch was set as 2. If the length of the standard motif is 9 bp and there is a pair of DnaA-boxes present, then the maximum mismatch is not more than either 2 (the more conserved DnaA-box) or 4 (the less conserved DnaA-box). If the length of the standard motif is more than 9 bp, the maximum mismatch of the sole (if singular) or the more conserved (if tandem) is increased at 3.
3. *Length of the spacer between the* *first located DnaA-box and the first DnaA-trios*. According to Supplementary Table S1, we assume that the longest spacer may include: the sequence between two DnaA-boxes (max 3 bp), a less conserved DnaA-box (generally 9 bp) and the second gap region previously named GC-rich region (4 bp on average). Therefore, the maximum predesigned length of the spacer is set to 16 bp, which is the sum of the three parts.
4. *Pattern of the DnaA-trios*. The base in the middle of each DnaA-trios trinucleotide motif is a conserved adenine. Analysis of the *B. subtilis* DnaA-trios suggested that a set of three motifs was minimally required for origin activity (22). Therefore, the general pattern of DnaA-trios was set as (.A.){3,}, which means no less than three motifs of NAN (N is any base) is allowed.

The searching process is divided into two steps (Supplementary Figure S2B). First, the program searches candidate sequence with the general pattern of the BUS sequence (Supplementary Figure S2D). In this step, it locates a DnaA-box (the first located DnaA-box) (mismatch ≤2) with a distance of 0–16 bp from the DnaA-trio. After, the second DnaA-box (mismatch ≤4) is searched for upstream or downstream of the DnaA-box identified in the first step. It should be noted that if this last DnaA-box is identified upstream of the first one and it appears to have less mismatches, the actual length of the spacer is counted from the second located DnaA-box and, therefore, will be more than 16 bp.

The screening process is divided into three steps (Supplementary Figure S2C). First, the program conserves the candidate sequences with the highest DnaA-trios score. Then, the candidate sequences containing the minimum number of mismatches in the DnaA-box (the sole if singular or the more conserved if tandem) are retained. After these two steps >94% of the genomes have a single candidate sequence. Third, the candidate sequences with the spacer length closest to 13 base pairs are retained, since it is the length of the most common spacer. After this step almost all (>99%) the genomes have a single candidate sequence left.

## RESULTS AND DISCUSSION

A bioinformatic analysis was performed to search for the BUS sequence motifs within chromosome origins. *oriC* sequences from bacterial genomes currently available in GenBank have been predicted using a method comprising the Z-curve analysis for nucleotide distribution asymmetry, DnaA-box distribution, genes adjacent to candidate *oriC* and phylogenetic relationships (DoriC database http://tubic.org/doric) (25). A search program was designed to recognize the potential BUS sequences, which required at least three putative DnaA-trios motifs (3′-NAN-5′) adjacent to (≤16 base pairs) a DnaA-box within each origin region (Supplementary Figure S2, Table S2). This program interrogated the predicted *oriC* on the primary chromosome of 2070 species and identified 1755 origins (85%) to contain potential BUS sequences (Supplementary Tables S1, Supplementary Figure S2).

Investigation of predicted BUS sequences from nine phyla containing at least fifteen representative sample genomes suggested that the DnaA-trio sequence 3′-GAT-5′ is the most frequent, particularly at the position proximal to the DnaA-box (Supplementary Figures S3 and S4). The most frequent arrangement of DnaA-boxes (57%) was predicted to be a tandem set where the upstream site tended to be a closer match to the consensus sequence (Supplementary Figure S3) (note that this may be an underestimation, as DnaA can bind to low affinity DnaA-boxes that deviate from high affinity sites (26)). Thespacer between the more highly conserved DnaA-box (or the sole site if only one was predicted) and DnaA-trios showed a peak around 10–13 base pairs and appeared to be a maximum distance allowed between the DnaA-box and DnaA-trios (Supplementary Figure S5). Interestingly, overlaying the properties of the putative unwinding modules around a highly resolved tree of life (27) reveals that they are distributed throughout the bacterial domain (Figure 1C). This broad conservation suggests that the BUS sequences identified in *B. subtilis* could be an ancient feature of bacterial chromosomes.

Based on the bioinformatic analysis described above, we next sought to directly test whether the basal *oriC* unwinding mechanism was functionally conserved in species across the bacterial domain. Previously, dsDNA unwinding activity of *B. subtilis* DnaA on its own minimal replication origin was reconstituted *in vitro* using purified proteins and short oligonucleotide scaffolds, where the reaction products were separated by size on a polyacrylamide gel (2). To improve both the temporal resolution and the throughput of this DnaA unwinding reaction, a fluorescence assay was developed. For this, two adjacent oligonucleotides in the scaffold were labelled with distinct molecular probes: the upstream oligonucleotide contained a Black Hole Quencher (BHQ2) at the 3′ terminus while the downstream oligonucleotide contained a Cy5 fluorophore at the 5′ terminus, thus juxtaposing the two probes (Figure 2A). When the assembled scaffold is intact, BHQ2 absorbs the energy emitted from Cy5 and low levels of fluorescence are produced. However, if DnaA can separate the scaffold strands, the liberated Cy5-labelled oligonucleotide emits a detectable signal (Figure 2A, B).

Fluorescence was observed when wild-type *B. subtilis* DnaA was incubated with ATP (Figure 2B), consistent with previous work (2,17). Reaction samples run on a polyacrylamide gel confirmed that the fluorescent signal was generated by specific strand detachment (Figure 2B) and that >80% of the scaffolds were being separated (Supplementary Figure S6). Reactions performed using either wild-type DnaA with ADP or DnaA^R264A^ (arginine finger substitution) with ATP did not produce a fluorescent signal, suggesting that DnaA oligomerization was required for unwinding (Figure 2B, C). Also, reactions performed using DnaA^I190A^ (ssDNA binding substitution within the ISM) with ATP did not produce a fluorescent signal, indicating that ssDNA binding activity is necessary for unwinding (Figure 2C). Critically, DNA scaffolds in which the sequences of the DnaA-boxes or DnaA-trios were mutagenized to impair DnaA binding (DnaA-box^MUT^ and DnaA-trios^MUT^) (2,28) did not produce a fluorescent signal, demonstrating that these sequence elements are required for the observed DnaA-dependent strand separation activity (Figure 2D). Interestingly, although *B. subtilis* contains a bona-fide set of tandem DnaA-boxes (22), only the upstream DnaA-box was required for strand separation (Figure 2D) while the downstream DnaA-box lowers the effective DnaA concentration required for strand separation (Supplementary Figure S7). These results are consistent with genetic analysis of these DnaA-boxes *in vivo* and they support the bioinformatic analysis where BUS modules with only a single DnaA-box were identified (22) (Supplementary Figure S3).

Having established an assay for *oriC* specific unwinding, we next cloned, expressed and purified DnaA homologs from a broad range of species (e.g. Gram-positive and Gram-negative) where BUS sequences were predicted: *Staphylococcus aureus*, *Listeria monocytogenes*, *Enterococcus faecalis*, *Helicobacter pylori*, *Synechococcus elongatus* and *Deinococcus radiodurans*. These organisms were selected either because they represent pathogenic species relevant to human health (*S.a./L.m./E.c./ H.p*.), because of their importance to environmental ecology (*S.e*) (29), or because they are thought to represent an ancestral bacterial lineage (*D.r*.) (30–32). All DnaA homologs were engineered to contain both an N-terminal FLAG_3_ epitope to facilitate immunoblotting and a pair of cysteine substitutions to allow crosslinking of DnaA proteins. The FLAG-tag and cysteine substitutions did not significantly affect the activities of *B. subtilis* DnaA *in vitro* (Supplementary Figure S8).

**Figure 3.**
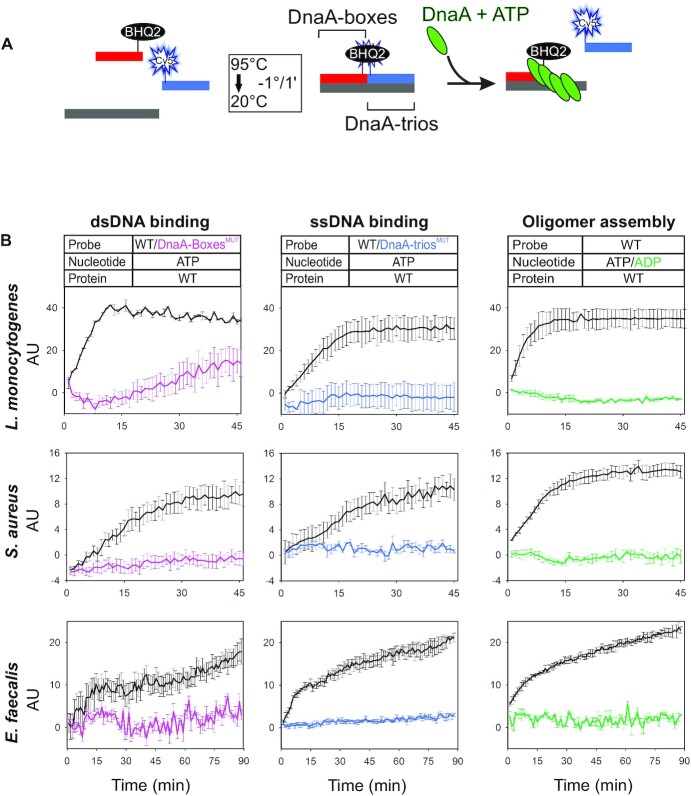
Diverse Gram-positive species have a functional BUS. (**A**) Schematic representation of the BHQ-SSA. (**B**) BHQ-SSA using Gram-positive DnaA homologs indicates that DnaA-boxes (left column), DnaA-trios (central column) and ATP (right column) are required for scaffold unwinding. Data represent the mean and standard deviation from three independent experiments. Y-axis shows fluorescence arbitrary unit (AU).

**Figure 4.**
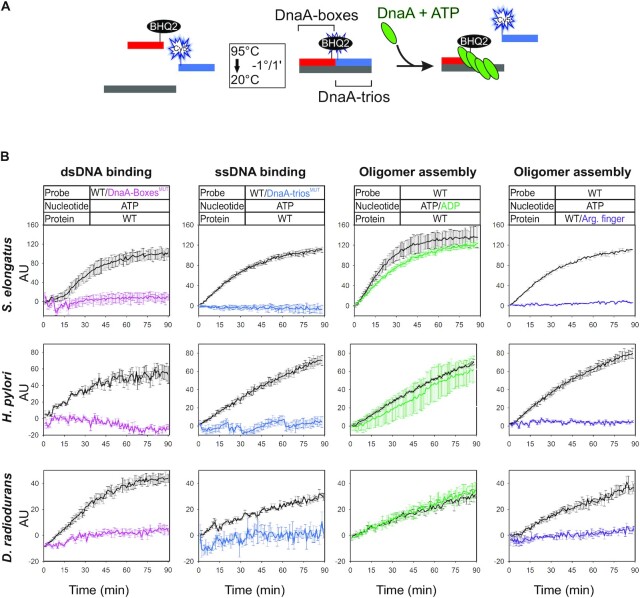
Diverse Gram-negative species have a functional BUS. (**A**) Schematic representation of the BHQ-SSA. (**b**) BHQ-SSA using Gram-negative DnaA homologs indicates that DnaA-boxes (first column), DnaA-trios (second column) and the arginine finger (*Se*DnaA^R261A^, *Hp* DnaA^R298A^, *Dr* DnaA^R264A^, fourth column) are required for scaffold unwinding, whereas ATP is not (third column). Data represent the mean and standard deviation from three independent experiments. Y-axis shows fluorescence arbitrary unit (AU).

Each DnaA homolog was incubated with DNA scaffolds containing the cognate DnaA-boxes and DnaA-trios from the respective chromosome origin. All DnaA homologs demonstrated unwinding activity on their native substrate, showing an increase in fluorescence over time (Figures 3B and 4B). Critically, in all cases the observed unwinding activity required the presence of the DnaA-boxes and DnaA-trios, indicating that both elements of the BUS are crucial for the system (Figures 3B and 4B).

To investigate the strand separation mechanism, reactions were performed in the presence of ADP to disfavour oligomerization. Under this condition all of the Gram-positive DnaA homologs (*S. aureus*, *L. monocytogenes, E. faecalis*) were unable to unwind the DNA strands, consistent with the model that ATP-dependent oligomer formation is required (Figure 3B). To further probe the BUS of these organisms, we assessed the ability of each DnaA homolog to assemble into an oligomer specifically on scaffolds contain BUS sequence elements. To capture DnaA oligomers, proteins were incubated with various scaffolds and then crosslinked using bismaleimidoethane (BMOE), which specifically reacts with the cysteines that were genetically engineered into the AAA+ domain of the recombinant DnaA proteins (Supplementary Figures S8 and S9) (22,33). DnaA homologs were then separated on a polyacrylamide gel and visualized by immunoblotting (Supplementary Figure S9). In all cases oligomer formation required ATP, DnaA-boxes and a 5′-tail encoding DnaA-trios (Supplementary Figure S9). Additionally, for *S. aureus* DnaA, a ssDNA binding variant (DnaA^I195A^) was analyzed and found defective in oligomer formation (Supplementary Figure S9). The results indicate that for Gram-positive DnaA homologs, their respective DnaA-trios specify oligomerization on ssDNA.

In contrast, all Gram-negative homologs (*H. pylori*, *S. elongates, D. radiodurans*) were competent to separate the DNA strands in the presence of ADP, at rates that were similar to the reaction with ATP (Figure 4B). While the interaction between the γ-phosphate of ATP and the arginine finger establishes an important electrostatic interaction between two DnaA proteins in an oligomer, additionally there is an extensive interface formed between adjacent AAA+ motifs (1005 Å buried surface area) (34). Moreover, it has been observed in *E. coli* that a lethal *dnaA* mutant producing a DnaA variant defective in oligomerization can be rescued by compensatory mutations in DnaA-boxes (35). These observations suggest that multiple interactions may be involved in establishing an active DnaA nucleoprotein complex, and the relative importance of each component may vary depending upon the bacterial species.

Therefore, to investigate whether a protein oligomer may be required for strand displacement activity of the Gram-negative DnaA homologs, we purified variants with the arginine finger residue replaced with alanine, as this key amino acid plays roles both contacting ATP *in trans* and also stabilizing the AAA+ fold *in cis* (11). Here, all DnaA homologs lacking the arginine finger were defective in strand separation activity, consistent with the notion that this residue is required to fulfil DnaA activity (Figure 4B). To assess the integrity of the arginine finger variants, their ability to bind the fluorescent nucleotide analog TNP-ATP was measured (36). In the presence of *B. subtilis* DnaA, TNP-ATP exhibits an increase in fluorescence intensity that can be competed away by addition of unlabelled ATP, indicating that TNP-ATP is binding within the nucleotide binding pocket (Supplementary Figure S10A, B). For all DnaA arginine finger mutants tested, the increase in TNP-ATP fluorescence was comparable to the wild-type homolog (Supplementary Figure S10C-F). Taken together, these observations indicate that DnaA homologs lacking the arginine finger residue are properly folded, consistent with the model that the defect in strand separation is due to disruption of protein-protein interactions. However, both the inability to detect crosslinking between AAA+ domains of Gram-negative DnaA homologs and the observation that these proteins are active in the presence of ADP suggests that these nucleoprotein complexes may be distinct from their Gram-positive counterparts.

Finally, to test the link between specific DnaA-trio binding activity and DNA strand separation, ssDNA binding mutants were purified (*S. aureus* DnaA^I195A^, *S. elongatus* DnaA^I224A^, *H. pylori* DnaA^L187A^, *D. radiodurans* DnaA^I190A^). Comparing these variants to the wild-type proteins, a complete loss of strand separation activity was observed (Supplementary Figure S11). All mutant DnaA homologs were able to bind TNP-ATP similarly to their respective wild-type protein, indicating that they are correctly folded (Supplementary Figure S10). Taken together, the data suggests that each DnaA homolog, interacting with its cognate set of DnaA-boxes and DnaA-trios, constitutes an unwinding system with many of the features that have been described for the *B. subtilis* BUS (2).

**Figure 5.**
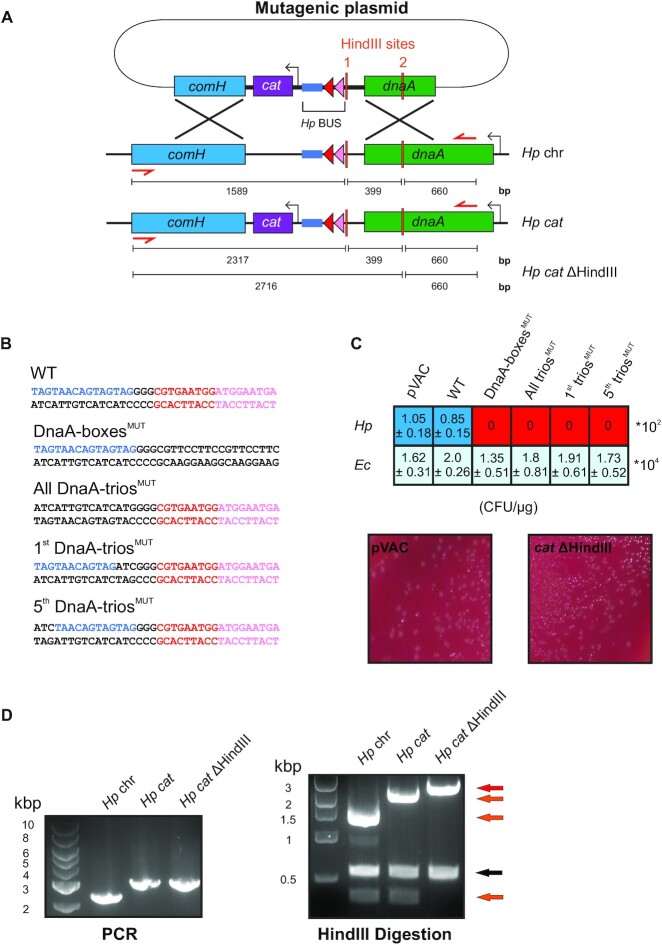
The BUS sequence elements are essential for the viability of *H. pylori*. (**A**) Method used for allelic exchange of *H. pylori oriC* sequences. External primer pairs used to amplify the chromosomal DNA are shown with red arrows and the sizes of possible DNA fragment resulting from HindIII endonuclease cleavage are indicated. (**B**) Mutations created within BUS sequences using the plasmid integration vector. (**C**) Table representing the number of colony forming units obtained with each mutagenic plasmid in either *H. pylori* or *E. coli*. An example of *H. pylori* transformants obtained using plasmid with the wild-type BUS sequence elements is reported below the table. (**D**) Agarose gel showing the BUS PCR products prior and after digestion with HindIII. All reactions contained a 660 bp fragment (black arrow). When two HindIII sites are present, as in wild-type, then two additional DNA fragments are produced (orange arrows). Upon mutagenesis of HindIII site #1, only a single additional DNA fragment is observed (red arrow).

While the reconstitution of DnaA unwinding activity suggests a conserved mechanism, the physiological relevance of the BUS for DNA replication initiation in other organisms remained unknown. Therefore, we developed a genetic approach to test for the importance of the BUS sequences within the *oriC* of *H. pylori*, an experimentally tractable Gram-negative bacterial pathogen distantly related to *B. subtilis* with a defined chromosome origin (37). The *H. pylori* BUS elements along with flanking sequences were cloned into a vector for allelic exchange of *oriC*, and an antibiotic resistance cassette was integrated immediately downstream of the unwinding region to ensure high genetic linkage with mutations (Figure 5A). Site-directed mutagenesis was used to disrupt each sequence element of the *H. pylori* BUS (Figure 5B). The transformation efficiency achieved with this *oriC* vector was similar to a control plasmid that integrates at the non-essential *vacA* locus, indicating that the antibiotic resistance cassette was not impairing origin function (Figure 5C). Moreover, using this *oriC* vector it was possible to create a strain containing mutation in a HindIII restriction enzyme site located adjacent to the BUS sequence elements (Figure 5D). This shows that mutagenesis within the *H. pylori* unwinding region is possible, as long as non-essential sequences are being targeted.

Mutations in the DnaA-boxes and DnaA-trios of the *oriC* vector were created and used to transform *H. pylori* (Figure 5B). While the parental plasmid readily produced transformants (∼100 CFU/μg DNA), none were detected when *oriC* vectors containing mutated BUS elements (Figure 5C, Supplementary Figure S12). In parallel each plasmid was transformed into *E. coli*, where all produced transformants at a similar efficiency, indicating that the integrity of the various DNA substrates was sound (Figure 5C). Taken together, the results suggest that the BUS sequence elements within the *H. pylori* chromosome origin are functional and critical for DNA replication initiation.

In summary, we performed a bioinformatic analysis of bacterial replication origins to search for BUS sequence elements (DnaA-trios adjacent to a DnaA-box), leading to the prediction that this module is widespread in chromosomes throughout the bacterial domain. We went on to purify several DnaA homologs from diverse species and found that the ability to unwind their cognate replication origins is dependent upon DnaA-boxes, DnaA-trios and the ability of DnaA to form an oligomer that specifically binds ssDNA. The functional conservation and broad distribution of the BUS elements lead us to suggest that they compose part of an ancestral bacterial chromosome origin unwinding module.

DnaA-trios were not identified in 15% of the bacterial genomes interrogated. This could be due to deviations from the established architecture (DnaA-box, spacer, DnaA-trios) or sequences (DnaA-box or DnaA-trios) used to construct the search algorithm. For example, based on the precedent of DnaA-box divergence (38,39), we suspect that there could be deviant DnaA-trio motifs (e.g. without the adenine at the middle position). Alternatively, it is established that some bacteria have naturally evolved distinct pathways for initiating DNA replication, with the extreme situation being completely independent of DnaA (40–42). In such cases, DnaA-trios would not be expected to be retained at *oriC*. It is interesting to note that the *Escherichia coli* chromosome origin, which has been a paradigm for studying bacterial DNA replication initiation, appears to have undergone a major rearrangement as *oriC* is separated from *dnaA* (20). The BUS score for *E. coli* was relatively weak, suggesting that its replication initiation mechanism may have diverged from the ancestral system.

Looking forward, antibiotics with novel modes of action are urgently required to combat the growing threat posed by multi-drug resistant bacteria. No antibiotics in clinical use act directly on the bacterial DNA replication machinery; DNA synthesis is thus an underexploited drug target (43). Because DnaA is an essential protein, conserved in pathogens, and distinct from the eukaryotic initiation system, the basal bacterial replication initiation reaction with its multiple protein:DNA and protein:protein interactions is an attractive target for drug development (44). We note that the BUS unwinding assay described here can be used to screen for small molecules that inhibit DnaA homologs.

## DATA AVAILABILITY

*oriC* sequences used to search BUS sequences are available from theDoriC database (http://tubic.org/doric). The predicted BUS sequences are shown in Supplementary Table S2.

## Supplementary Material

gkab560_Supplemental_Files
