# Supplementary material for: Evidence for a chromosome origin unwinding system broadly conserved in bacteria
Source: Nucleic Acids Res. 2021 Jul 1;49(13):7525–36. doi: 10.1093/nar/gkab560 (PMC8287927; doi:10.1093/nar/gkab560)
Supplement: gkab560_Supplemental_Files [file gkab560_supplemental_files.zip › Pelliciari_Figures_SUPP.pdf]

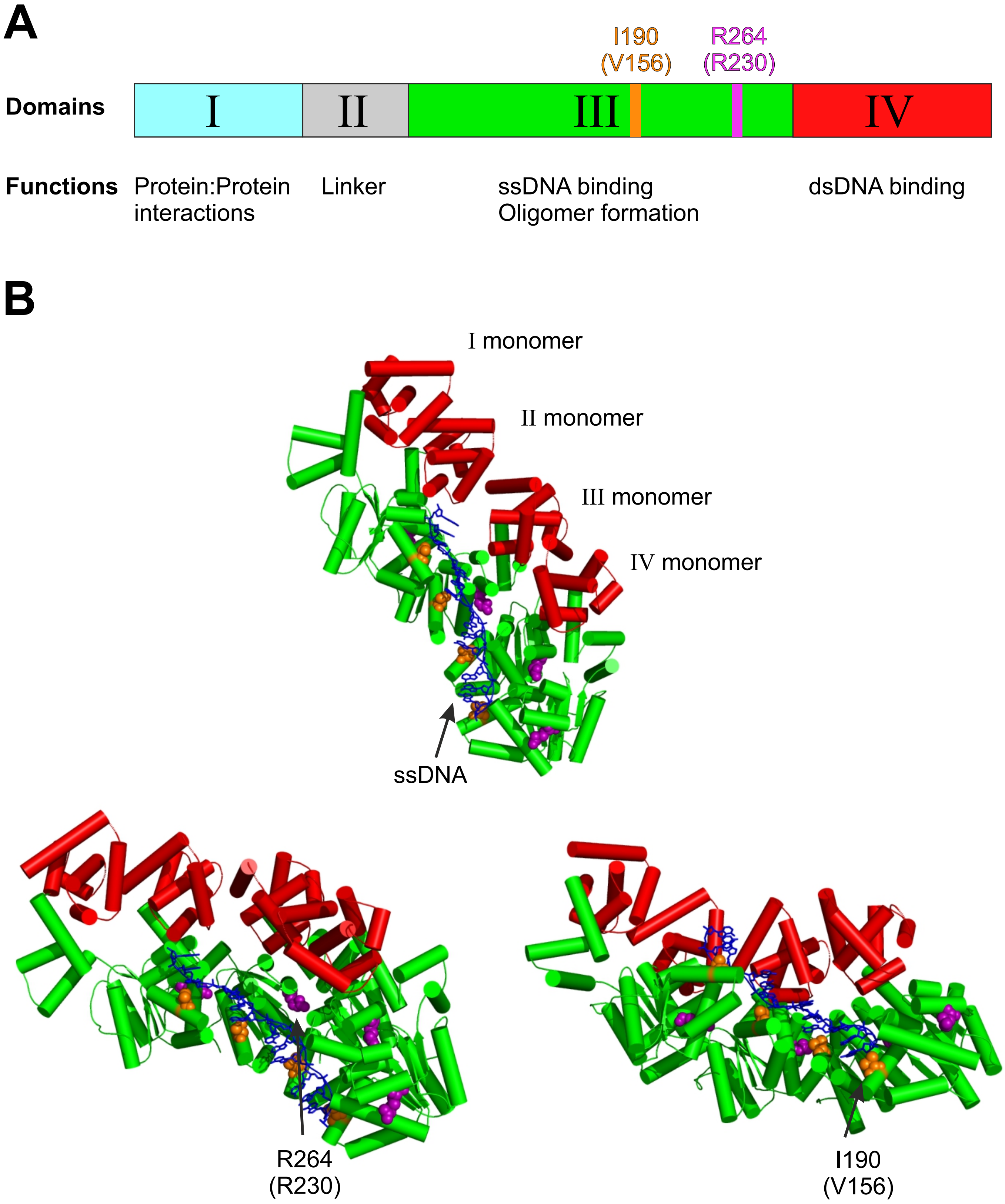
A
I190
(V156)
R264
(R230)
Domains
I
II
III
IV
Functions
Protein:Protein interactions
Linker
ssDNA binding
Oligomer formation
dsDNA binding
B
I monomer
II monomer
III monomer
IV monomer
ssDNA
R264
(R230)
I190
(V156)

Figure S1

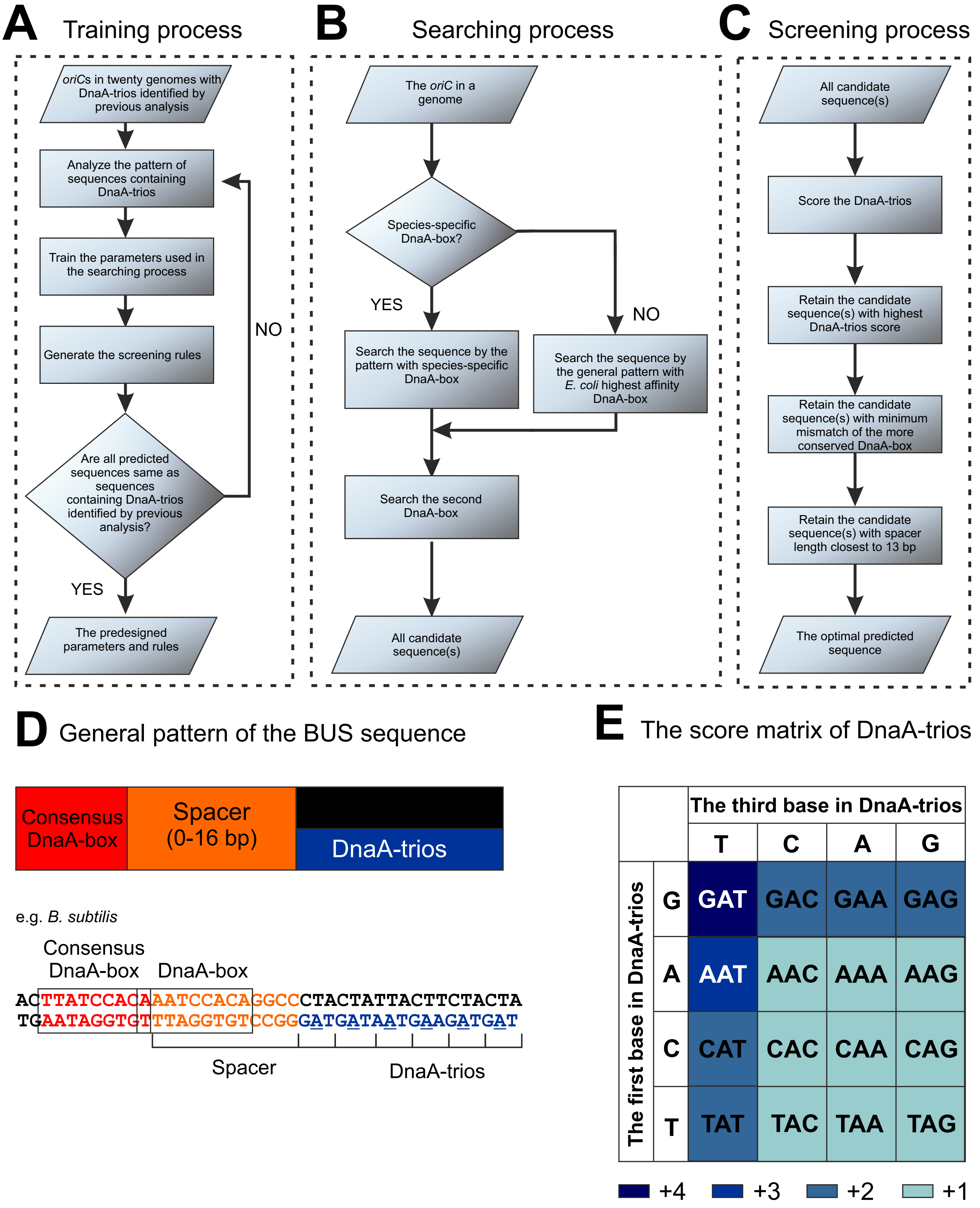
A Training process
oriCs in twenty genomes with DnaA-trios identified by previous analysis
Analyze the pattern of sequences containing DnaA-trios
Train the parameters used in the searching process
Generate the screening rules
Are all predicted sequences same as sequences containing DnaA-trios identified by previous analysis?
NO
YES
The predesigned parameters and rules
B Searching process
The oriC in a genome
Species-specific DnaA-box?
YES
NO
Search the sequence by the pattern with species-specific DnaA-box
Search the sequence by the general pattern with E. coli highest affinity DnaA-box
Search the second DnaA-box
All candidate sequence(s)
C Screening process
All candidate sequence(s)
Score the DnaA-trios
Retain the candidate sequence(s) with highest DnaA-trios score
Retain the candidate sequence(s) with minimum mismatch of the more conserved DnaA-box
Retain the candidate sequence(s) with spacer length closest to 13 bp
The optimal predicted sequence
D General pattern of the BUS sequence
Consensus DnaA-box
Spacer (0-16 bp)
DnaA-trios
e.g. B. subtilis
Consensus DnaA-box
DnaA-box
ACTTATCCACAAATCCACAGGCCCTACTATTACTTCTACTA
TGAATAGGTGTTTAGGTGTCCGGGATGATAATGAAGATGAT
Spacer
DnaA-trios
E The score matrix of DnaA-trios
The third base in DnaA-trios
T
C
A
G
The first base in DnaA-trios
G
GAT
GAC
GAA
GAG
A
AAT
AAC
AAA
AAG
C
CAT
CAC
CAA
CAG
T
TAT
TAC
TAA
TAG
+4
+3
+2
+1

Figure S2

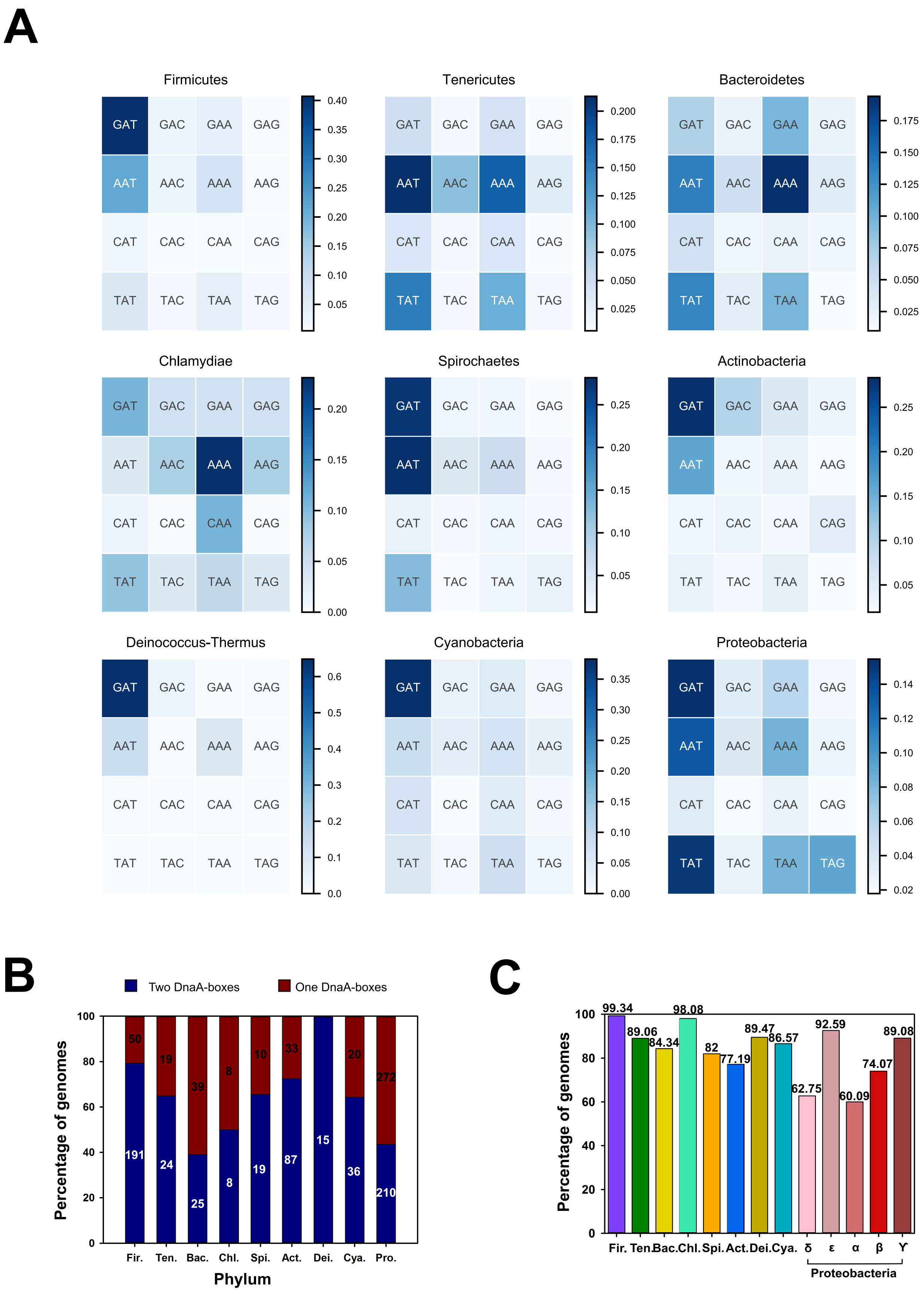
A
Firmicutes
Tenericutes
Bacteroidetes
Chlamydiae
Spirochaetes
Actinobacteria
Deinococcus-Thermus
Cyanobacteria
Proteobacteria
GAT GAC GAA GAG
AAT AAC AAA AAG
CAT CAC CAA CAG
TAT TAC TAA TAG
B
Two DnaA-boxes
One DnaA-boxes
Percentage of genomes
Phylum
Fir. Ten. Bac. Chl. Spi. Act. Dei. Cya. Pro.
50 19 39 8 10 33 20 272
191 24 25 8 19 87 15 36 210
C
Percentage of genomes
99.34 89.06 84.34 98.08 82 77.19 89.47 86.57 62.75 92.59 60.09 74.07 89.08
Fir. Ten. Bac. Chl. Spi. Act. Dei. Cya. δ ε α β ϒ
Proteobacteria

Figure S3

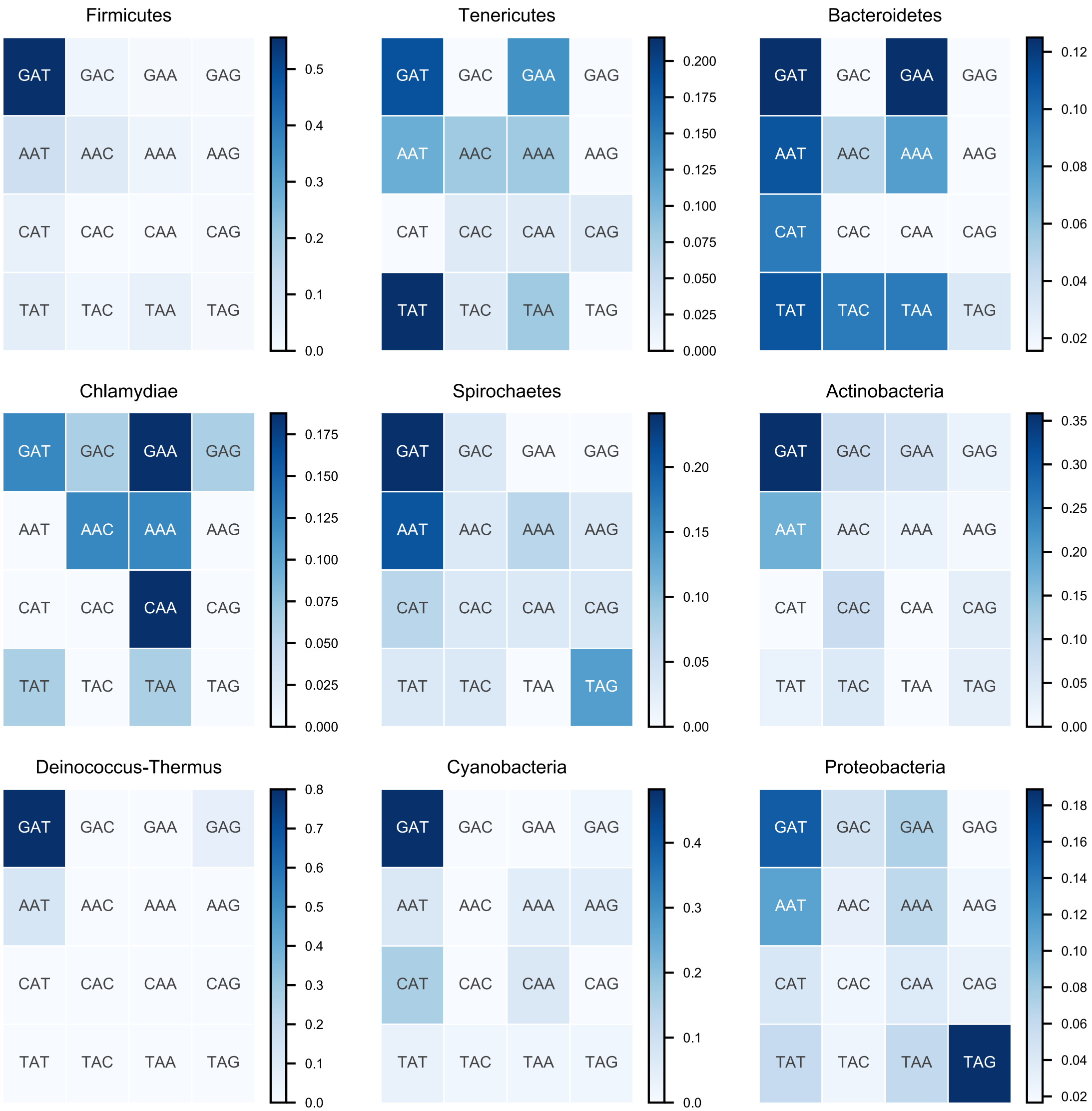
Firmicutes
Tenericutes
Bacteroidetes
Chlamydiae
Spirochaetes
Actinobacteria
Deinococcus-Thermus
Cyanobacteria
Proteobacteria
GAT GAC GAA GAG
AAT AAC AAA AAG
CAT CAC CAA CAG
TAT TAC TAA TAG

Figure S4

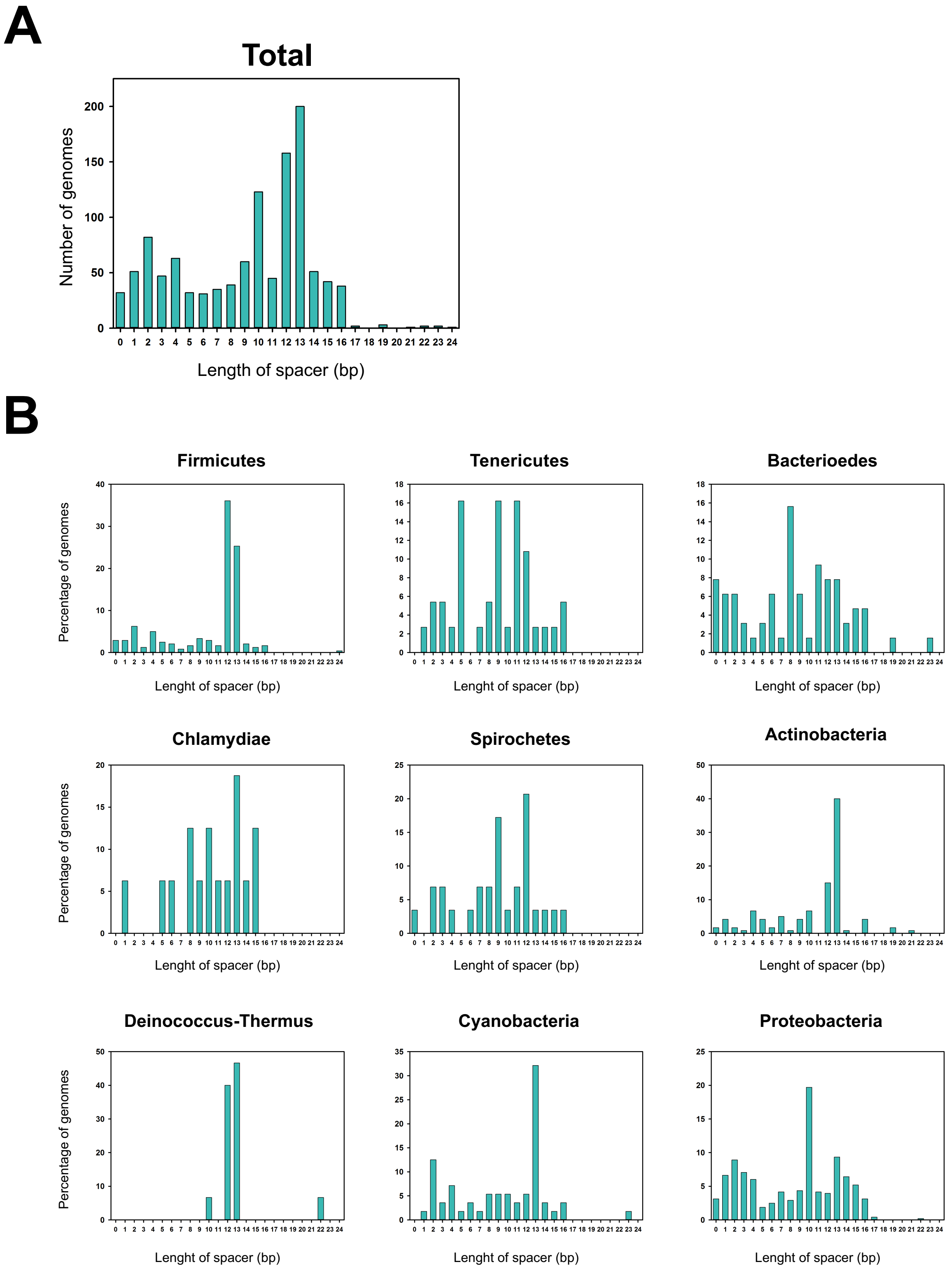
A
Total
Number of genomes
0
50
100
150
200
0 1 2 3 4 5 6 7 8 9 10 11 12 13 14 15 16 17 18 19 20 21 22 23 24
Length of spacer (bp)
B
Firmicutes
Tenericutes
Bacterioedes
Chlamydiae
Spirochetes
Actinobacteria
Deinococcus-Thermus
Cyanobacteria
Proteobacteria
Percentage of genomes
Lenght of spacer (bp)

Figure S5

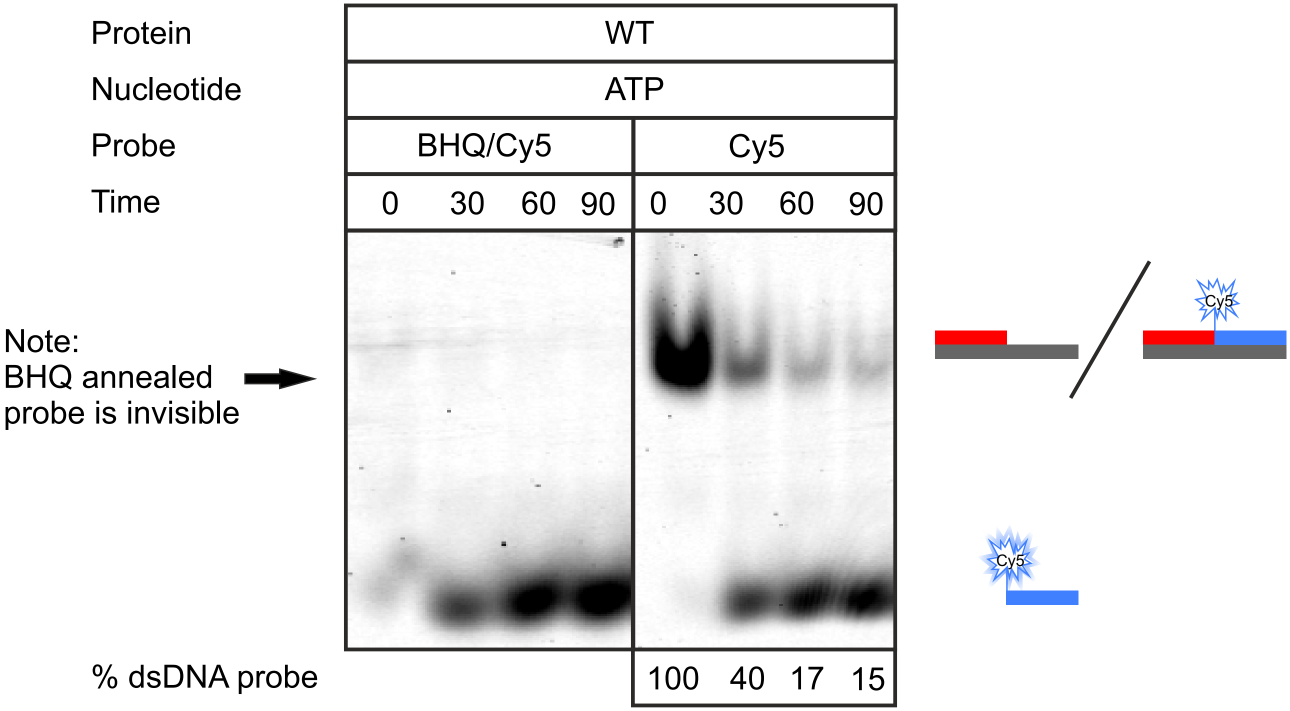
Protein
WT
Nucleotide
ATP
Probe
BHQ/Cy5
Cy5
Time
0 30 60 90
0 30 60 90
Note:
BHQ annealed probe is invisible
Cy5
Cy5
% dsDNA probe
100 40 17 15

Figure S6

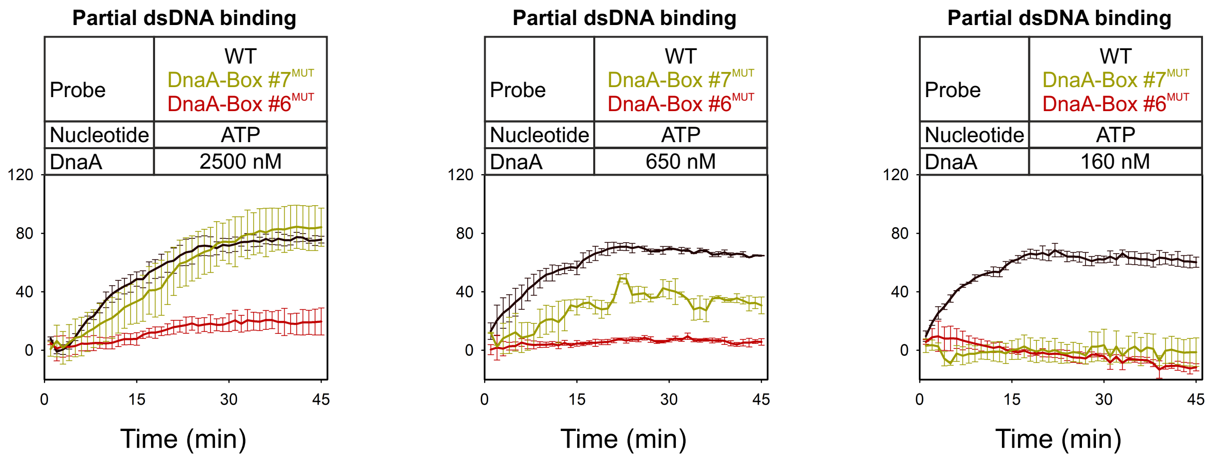
Partial dsDNA binding
Probe
WT
DnaA-Box #7MUT
DnaA-Box #6MUT
Nucleotide
ATP
DnaA
2500 nM
120
80
40
0
0
15
30
45
Time (min)
Partial dsDNA binding
Probe
WT
DnaA-Box #7MUT
DnaA-Box #6MUT
Nucleotide
ATP
DnaA
650 nM
120
80
40
0
0
15
30
45
Time (min)
Partial dsDNA binding
Probe
WT
DnaA-Box #7MUT
DnaA-Box #6MUT
Nucleotide
ATP
DnaA
160 nM
120
80
40
0
0
15
30
45
Time (min)

Figure S7

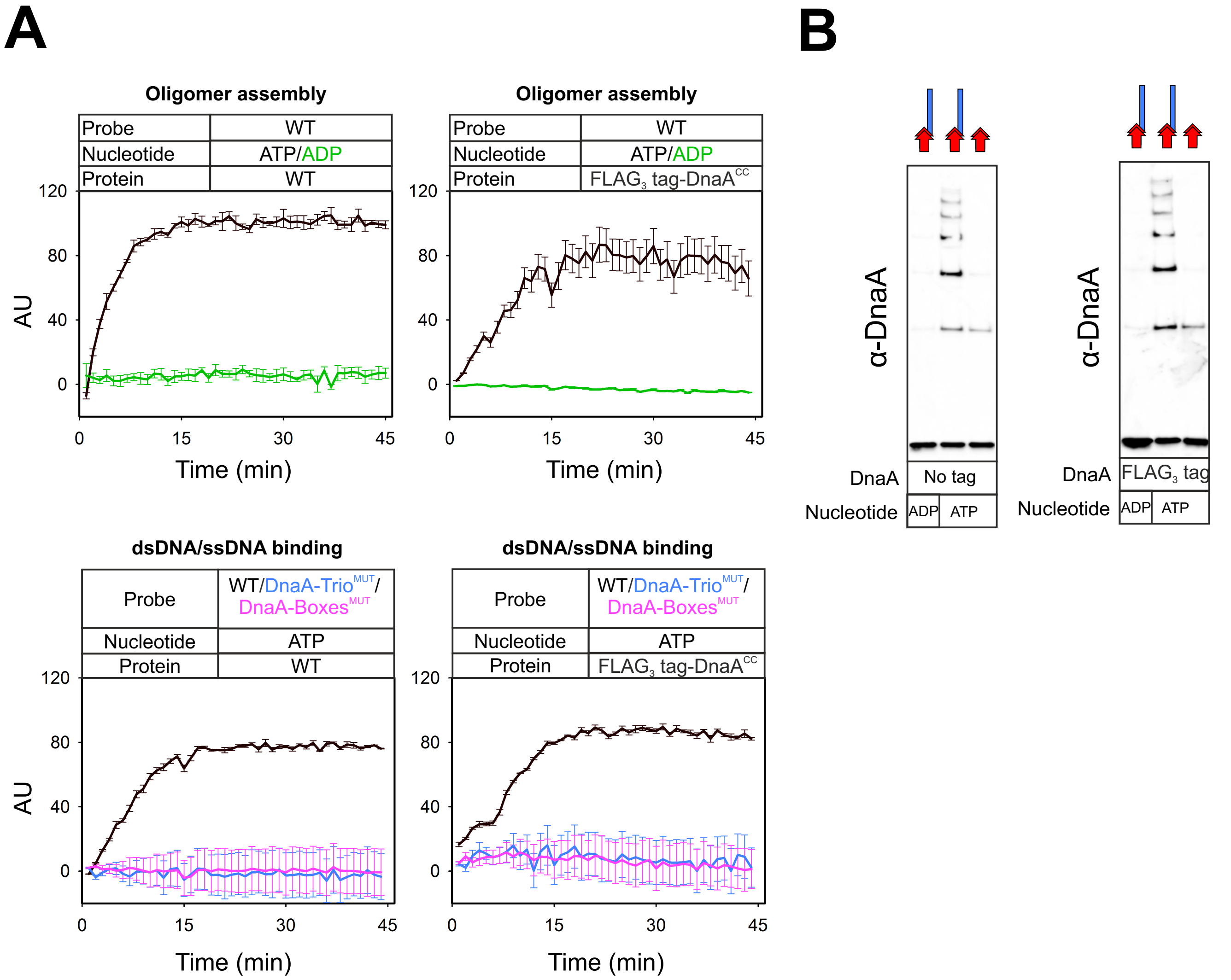
A
Oligomer assembly
Probe
WT
Nucleotide
ATP/ADP
Protein
WT
AU
Time (min)
Oligomer assembly
Probe
WT
Nucleotide
ATP/ADP
Protein
FLAG3 tag-DnaA^CC
Time (min)
dsDNA/ssDNA binding
Probe
WT/DnaA-Trio^MUT/ DnaA-Boxes^MUT
Nucleotide
ATP
Protein
WT
AU
Time (min)
dsDNA/ssDNA binding
Probe
WT/DnaA-Trio^MUT/ DnaA-Boxes^MUT
Nucleotide
ATP
Protein
FLAG3 tag-DnaA^CC
Time (min)
B
α-DnaA
DnaA
No tag
Nucleotide
ADP
ATP
α-DnaA
DnaA
FLAG3 tag
Nucleotide
ADP
ATP

Figure S8

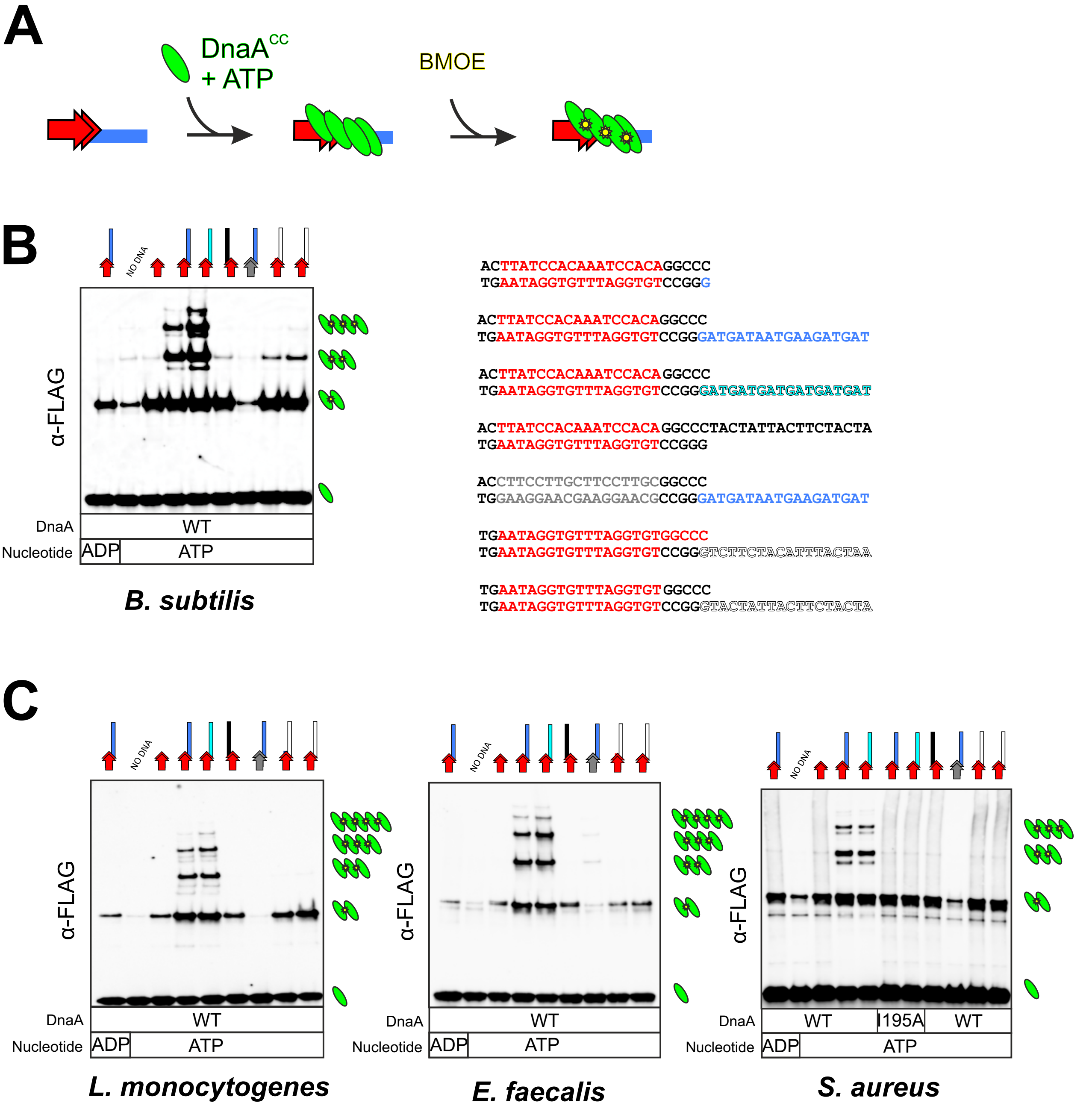

A
DnaA^cc
+ ATP
BMOE
B
NO DNA
α-FLAG
DnaA
WT
Nucleotide
ADP
ATP
B. subtilis
ACTTATCCACAAATCCACAGGCCC
TGAATAGGTGTTTAGGTGTCCGGG
ACTTATCCACAAATCCACAGGCCC
TGAATAGGTGTTTAGGTGTCCGGGATGATAATGAAGATGAT
ACTTATCCACAAATCCACAGGCCC
TGAATAGGTGTTTAGGTGTCCGGGATGATGATGATGATGAT
ACTTATCCACAAATCCACAGGCCCTACTATTACTTCTACTA
TGAATAGGTGTTTAGGTGTCCGGG
ACCTTCCTTGCTTCCTTGCGGCCC
TGGAAGGAACGAAGGAACGCCGGGATGATAATGAAGATGAT
TGAATAGGTGTTTAGGTGTGGCCC
TGAATAGGTGTTTAGGTGTCCGGGTCTTCTACATTTACTAA
TGAATAGGTGTTTAGGTGTGGCCC
TGAATAGGTGTTTAGGTGTCCGGGTACTATTACTTCTACTA
C
NO DNA
α-FLAG
DnaA
WT
Nucleotide
ADP
ATP
L. monocytogenes
NO DNA
α-FLAG
DnaA
WT
Nucleotide
ADP
ATP
E. faecalis
NO DNA
α-FLAG
DnaA
WT
I195A
WT
Nucleotide
ADP
ATP
S. aureus

Figure S9

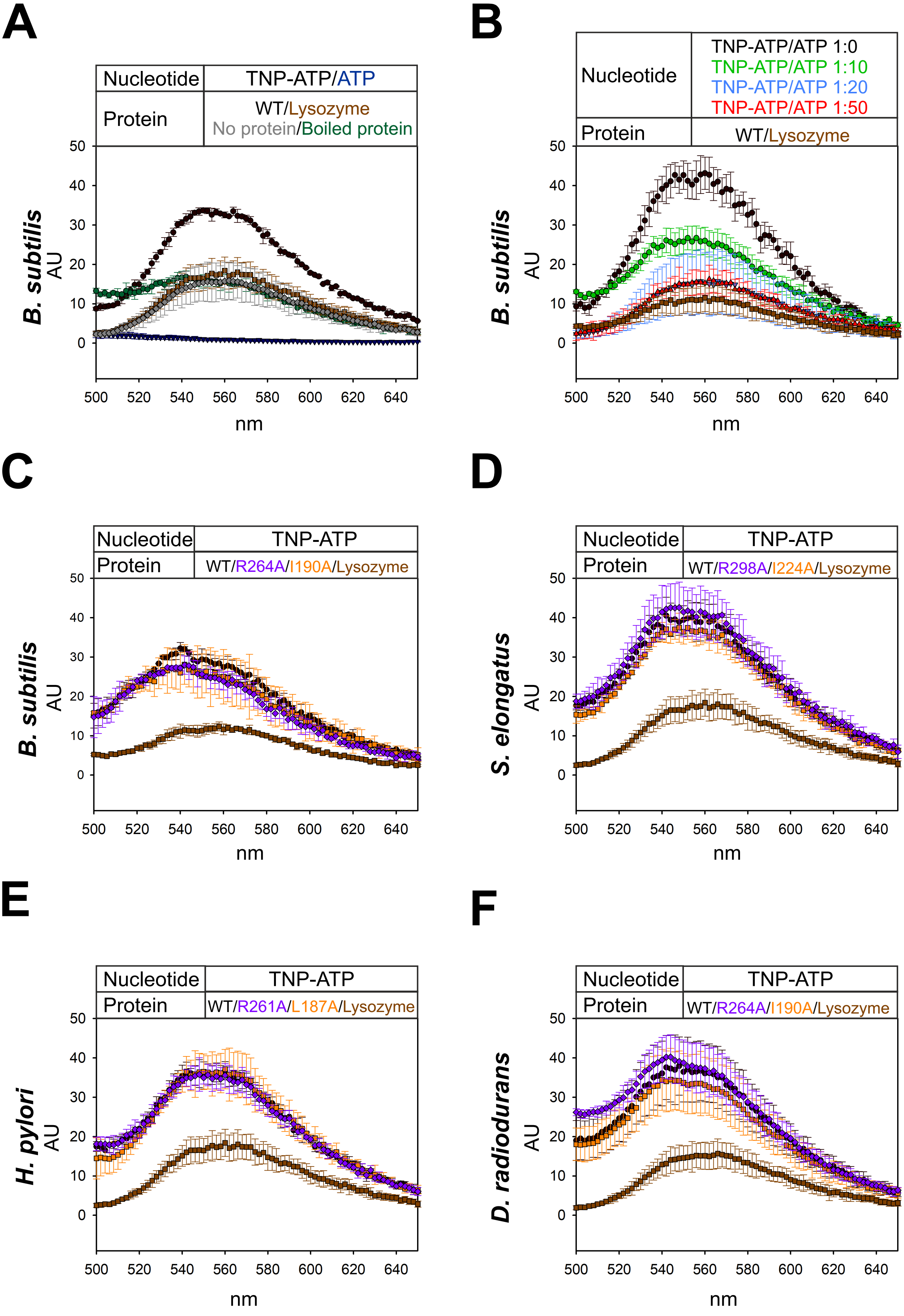

A
Nucleotide
TNP-ATP/ATP
Protein
WT/Lysozyme
No protein/Boiled protein
B. subtilis
AU
nm
B
Nucleotide
TNP-ATP/ATP 1:0
TNP-ATP/ATP 1:10
TNP-ATP/ATP 1:20
TNP-ATP/ATP 1:50
Protein
WT/Lysozyme
B. subtilis
AU
nm
C
Nucleotide
TNP-ATP
Protein
WT/R264A/I190A/Lysozyme
B. subtilis
AU
nm
D
Nucleotide
TNP-ATP
Protein
WT/R298A/I224A/Lysozyme
S. elongatus
AU
nm
E
Nucleotide
TNP-ATP
Protein
WT/R261A/L187A/Lysozyme
H. pylori
AU
nm
F
Nucleotide
TNP-ATP
Protein
WT/R264A/I190A/Lysozyme
D. radiodurans
AU
nm

Figure S10

ssDNA binding

Probe	WT
Nucleotide	ATP
Protein	WT/ssDNA mutant

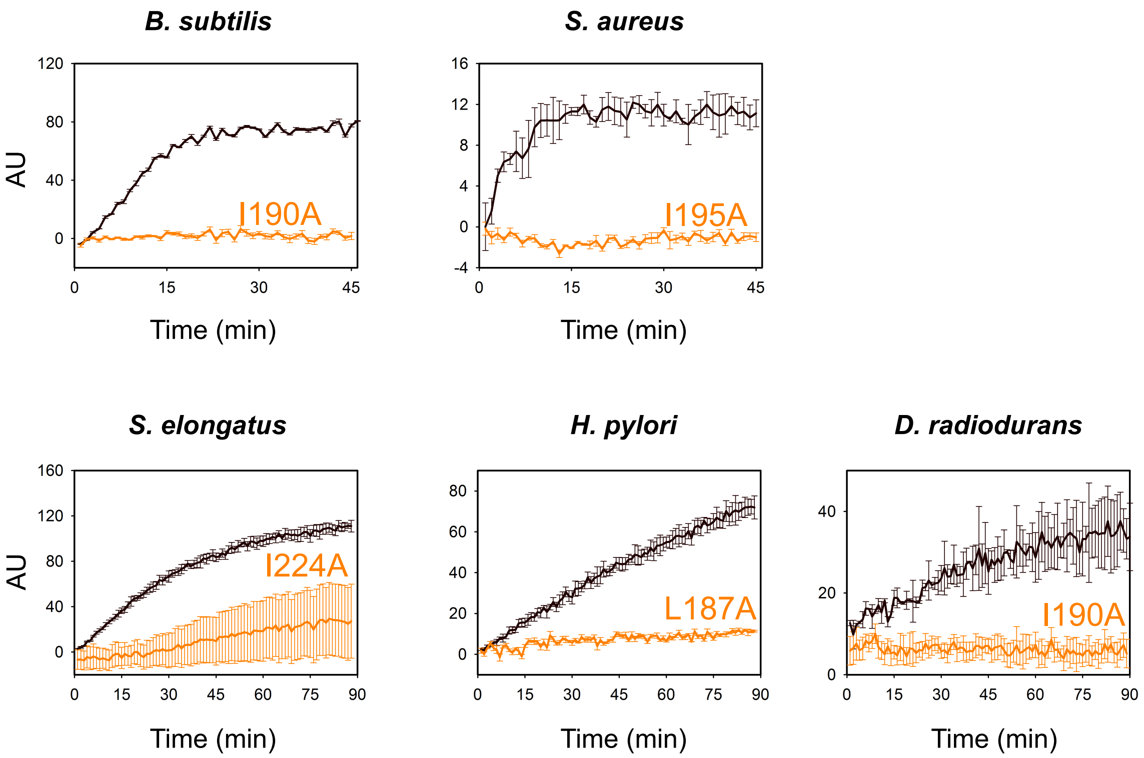

Figure S11

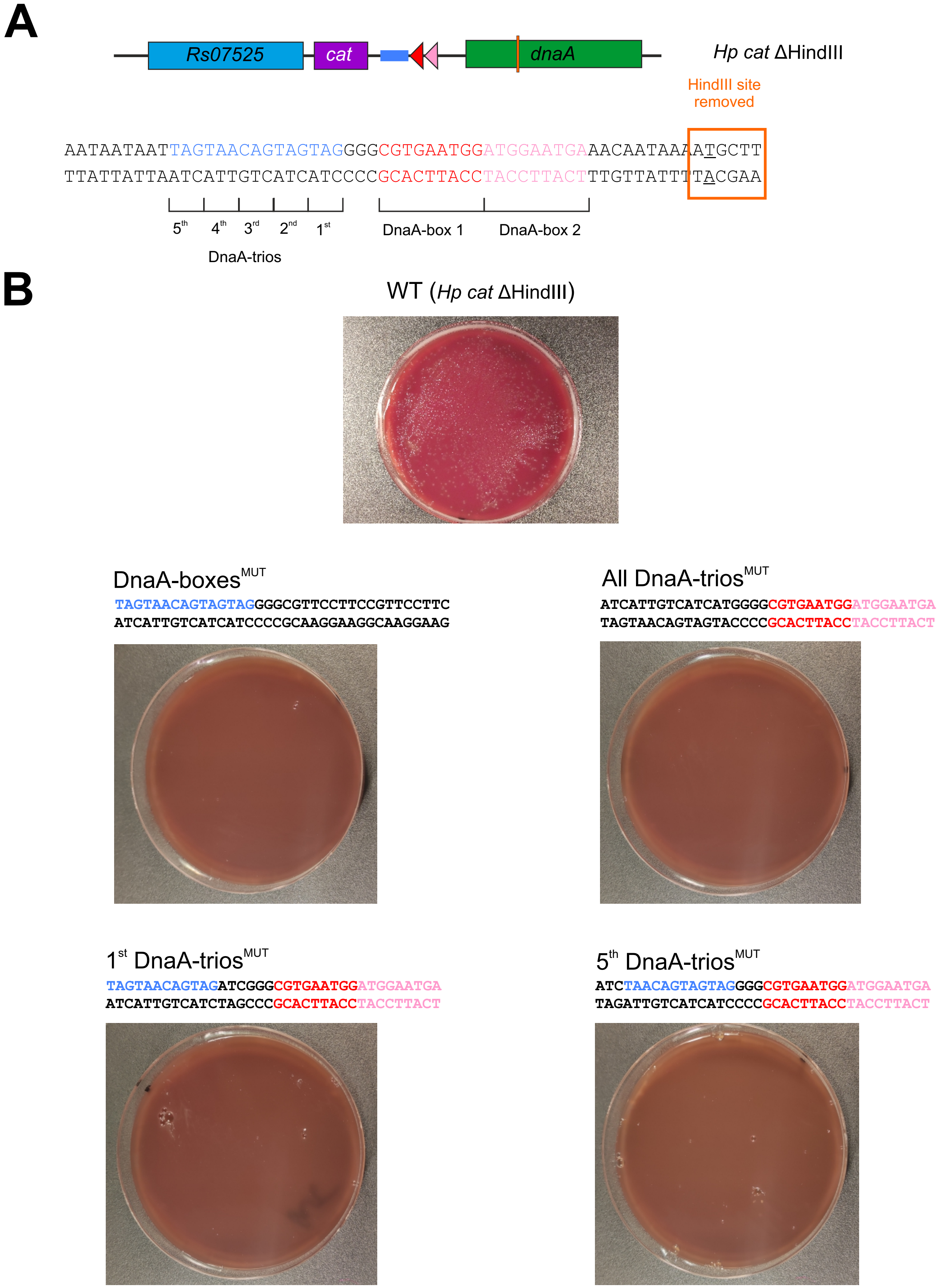
A
Rs07525
cat
dnaA
Hp cat ΔHindIII
HindIII site removed
AATAATAATTAGTAACAGTAGTAGGGGCGTGAATGGATGGAATGAAACAATAAAATGCTT
TTATTATTAATCATTGTCATCATCCCCGCACTTACCTACCTTACTTTGTTATTTTACGAA
5th 4th 3rd 2nd 1st
DnaA-trios
DnaA-box 1
DnaA-box 2
B
WT (Hp cat ΔHindIII)
DnaA-boxesMUT
TAGTAACAGTAGTAGGGGCGTTCCTTCCGTTCCTTC
ATCATTGTCATCATCCCCGCAAGGAAGGCAAGGAAG
All DnaA-triosMUT
ATCATTGTCATCATGGGGCGTGAATGGATGGAATGA
TAGTAACAGTAGTACCCCGCACTTACCTACCTTACT
1st DnaA-triosMUT
TAGTAACAGTAGATCGGGCGTGAATGGATGGAATGA
ATCATTGTCATCTAGCCCGCACTTACCTACCTTACT
5th DnaA-triosMUT
ATCTAACAGTAGTAGGGGCGTGAATGGATGGAATGA
TAGATTGTCATCATCCCCGCACTTACCTACCTTACT

Figure S12

Supplementary Figures

Figure S1. Structure of and function of DnaA. (a) Organization and functions of conserved DnaA domains. (**b**) Crystal structure of a DnaA oligomer engaging ssDNA (in blue). The monomers are shown in green and red to match the domain described above. The arginine finger (R264) that contacts the γ-phosphate of the ATP bound in nucleotide binding pocket of the adjacent monomer is shown in purple and the ssDNA binding residue (I190) is shown in orange. Indicated residues refer to *B. subtilis* DnaA (corresponding *A. aeolicus* in brackets).

Figure S2. Schematic diagram of algorithm designed to predict the BUS sequences within bacterial *oriC*. (a) The parameters and rules required by the program generated during the training process based on twenty putative BUS sequences (21). **(b)** Searching process of the candidate sequences of DnaA-trios in a genome. **(c)** Screening process of the optimal sequence in a genome. **(d)** General pattern of the BUS sequence. The DnaA-box is shown in red, the spacer in orange (which may or may not contain a second DnaA-box), and the DnaA-trios in blue. **(e)** The scoring matrix of DnaA-trios used during the screening process. Each trinucleotide motif is scored in turn, and the final score of DnaA-trios in each predicted sequence is the sum of these scores. This score matrix is designed based on the similarity of DnaA-trios to the standard DnaA-trios (3'-GAT-5').

Figure S3. Occurrence of possible BUS sequence motifs across nine phyla. (a) Heat maps displaying the frequency of DnaA-trio trinucleotide motifs across nine phyla including at least fifteen genomes with the predicted BUS sequences. The darker the colour, the higher the frequency of corresponding DnaA-trio. **(b)** Distribution across the nine phyla of BUS with either a single DnaA-box or a tandem set. The number of genomes analysed is indicated within the bar. **(c)** Histogram across the nine phyla showing the percentage of genomes predicted to contain BUS sequences.

Figure S4. The first DnaA-trio of a BUS. Heat maps displaying the frequency of the first DnaA-trio trinucleotide motif across nine phyla including at least fifteen genomes with the predicted BUS sequences. The darker the colour, the higher the frequency of corresponding DnaA-trio.

Figure S5. The spacer between a DnaA-box and the first DnaA-trio. Graphs displaying the frequency of the spacing between a DnaA-box (consensus if a tandem set) and the first DnaA-trio trinucleotide motif across nine phyla including at least fifteen genomes with the predicted BUS sequences.

Figure S6. BHQ-SSA efficiency. Polyacrylamide gel showing a comparison strand displacement assay between WT Cy5 and WT BHQ-Cy5 probe. The displacement rate between the two probes is very similar, as well as the overall efficiency that now can be calculated for the BHQ-Cy5 probe.

Figure S7. DnaA activity on DnaA-BOX#7^{MUT} is dependent on protein concentration. BHQ-SSA performed with different protein concentration on WT, DnaA-BOX#7^{MUT} and DnaA-BOX#6^{MUT} probe.

Figure S8. A FLAG-tag does not perturb *B. subtilis* DnaA activities *in vitro*. (a) BHQ-SSA performed under a range of conditions. In each case Flag$_3$-DnaA^{CC} activity was similar to DnaA^{CC}. Y axis represent fluorescence Arbitrary Unit (A.U.). **(b)** Crosslinking assay performed under a range of conditions. In each case Flag$_3$-DnaA^{CC} activity was similar to DnaA^{CC}.

Figure S9. BUS sequence elements promote DnaA filament formation. **(a)** Schematic representation of the experimental design. **(b)** Filament formation assay performed on *Bs*

$FLAG_3$-DnaA^{CC} and visualized by immunoblotting using α-FLAG antibody. The colour scheme of each scaffold represents a sequence/structure shown on the right. **(c)** Crosslinking assay performed with *Lm*, *Ef* and *Sa* $FLAG_3$-DnaA^{CC} homologs. The colours used to indicate the different DNA scaffolds follow the pattern used for *B. subtilis*, although the precise nucleotide sequences are the physiological ones (Table S3).

Figure S10. DnaA amino acid substitutions do not perturb ATP binding activity. (a) TNP-ATP binding experiment performed with wild-type *B. subtilis* DnaA. Lysozyme was used as a negative control for ATP binding (shown in brown in all experiments). **(b)** Competition assay between TNP-ATP and ATP using wild-type *B. subtilis* DnaA. **(c-f)** Experiments performed with wild-type DnaA homologs and variants (species indicated on the left side of each graph). In each case the wild-type protein are indicated in black, the arginine finger substitution in violet, and the ssDNA binding residue substitution in orange.

Figure S11. DnaA homologs with a key ssDNA binding residue substituted with alanine cannot unwind BUS scaffolds. BHQ-SSA performed on physiological substrates with either wild-type DnaA homologs (in black) or ssDNA binding mutants (*Bs* DnaA^{I190A}, *Sa* DnaA^{I195A}, *Se*DnaA^{I224A}, *Hp* DnaA^{L187A} and *Dr* DnaA^{I190A}). Y axis represent fluorescence Arbitrary Unit (A.U.).

Figure S12. *H. pylori* BUS sequences are essential *in vivo*. (a) On top is a schematic representation of the genomic region amplified to generate mutagenic plasmid for *H. pylori* BUS region. Shown below are the salient BUS sequence elements. The orange square demarks the HindIII cleavage site that has been mutagenized (underlined) in the template used for allelic exchange. **(b)** Representative images showing the results of *H. pylori* transformations. Nucleotide sequences showing mutations are provided.

Table S1. Predicted BUS sequences in DoriC 6.5.

Table S2. The training set of putative BUS sequences used to generate parameters and rules for the algorithm predicting BUSs sequence.

Table S3. Strain, oligonucleotides, plasmid, probes and synthetic genes used in this work.
